# The impact of sarcopenia and myosteatosis on postoperative outcomes in patients with inflammatory bowel disease

**DOI:** 10.1186/s41747-018-0072-3

**Published:** 2018-11-21

**Authors:** Stephen O’Brien, Richard G. Kavanagh, Brian W. Carey, Michael M. Maher, Owen J. O’Connor, Emmet J. Andrews

**Affiliations:** 1 The Hiram C. Polk Jr., MD Department of Surgery, Price Institute of Surgical Research, University of Louisville, Louisville, KY USA; 2 Department of Surgery, Cork University Hospital, Cork, Ireland; 3 Department of Radiology, Cork University Hospital, Cork, Ireland; 4 Alimentary Pharmabiotic Centre, University College Cork, Cork, Ireland

**Keywords:** Inflammatory bowel diseases, Sarcopenia, Muscle (skeletal), Morbidity, Length of stay, Tomography (x-ray, computed)

## Abstract

**Background:**

Inflammatory bowel disease (IBD) is a relatively common disorder with significant associated morbidity. Sarcopenia and myosteatosis are associated with adverse postoperative outcomes. This study investigated outcomes in IBD patients undergoing surgical resection relative to the presence of sarcopenia and myosteatosis.

**Methods:**

A retrospective analysis of a prospectively maintained surgical database was conducted. All patients undergoing elective or emergency resection for IBD between 2011 and 2016, with a contemporaneous perioperative computed tomography (CT) scan, were included. Patient demographics, clinical and biochemical measurements were collected. Skeletal muscle index and attenuation were measured on perioperative CT scans using Osirix version 5.6.1. Univariate and multivariate regression analysis was used to identify risk factors for adverse postoperative outcomes.

**Results:**

Seventy-seven patients (46 male, 31 female; mean age 42 years, range 20–80 years) were included. Thirty patients (30%) had sarcopenia and 26 (34%) had myosteatosis. Myosteatosis was significantly associated with increased hospital stay postoperatively (9 versus 13 days). Sarcopenia and myosteatosis were associated with hospital readmission within 30 days on univariate analysis. Multivariate regression analysis demonstrated an independent association between myosteatosis and hospital readmission. Sixteen patients (21%) had a clinically relevant postoperative complication, but an association with sarcopenia and myosteatosis was not observed. A neutrophil-lymphocyte ratio greater than 5 was predictive of clinically relevant postoperative complications on multivariate regression analysis.

**Conclusions:**

Myosteatosis was associated with increased hospital stay and increased 30-day hospital readmission rates on multivariate regression analysis. Sarcopenia and myosteatosis in IBD were not associated with clinically relevant postoperative complications.

## Key points

- Myosteatosis was associated with increased hospital stay and increased 30-day hospital readmission rates for patients who suffer from IBD requiring resection surgery
- The considerations of sarcopenia and myosteatosis as potential modifiable risk factors for adverse postoperative outcomes in patients who suffer from IBD requiring resection surgery merits further research

## Background

Inflammatory bowel disease (IBD) is a relatively common chronic inflammatory disorder of the gastrointestinal tract. Ulcerative colitis and Crohn disease are the principal subtypes. The incidence in Northern Europe is 6.3 per 100,000 person years for Crohn disease and 11.4 for ulcerative colitis [1, 2]. IBD has a complex pathophysiology consisting of an interaction between genetic susceptibility, the environment and the host immune responses.

Clinical differentiation between subtypes is essential with regard to management decisions. Up to 80% of patients with Crohn disease will require surgery in their lifetime and surgery is both an emergency and definitive therapy for ulcerative colitis [2, 3]. Up to 30% of patients with ulcerative colitis will require colectomy [2]. These patients suffer significant nutritional challenges associated with their pathology as a result of malabsorptive and chronic inflammatory states.

The incidence of postoperative complications in patients with IBD undergoing resection surgery ranges from 20 to 40% [4–6]. Morbidity associated with IBD may be partly attested to by the characteristics of the typical preoperative IBD patient: poor nutritional status, preoperative sepsis (localised or systemic) or intestinal obstruction. Often these patients will require a number of investigations in an attempt to delineate disease severity, disease activity, and the presence of complications in order to determine optimal management. Computed tomography (CT) is frequently used for this purpose [7]. CT can also be used to provide additional information pertaining to patients’ general health, including muscle volume and attenuation.

Sarcopenia has been defined by the European Working Group on Sarcopenia in Older People (EWGSOP) as a low muscle mass and either decreased muscle strength or low physical performance [8]. Interest in the role of sarcopenia in surgical patients has grown in the last number of years, especially in the field of oncology. A number of recent studies have demonstrated an adverse association between sarcopenia and immediate and long-term patient outcomes following surgery including postoperative complications, length of hospital stay, recurrence-free, and overall survival [9–11]. Myosteatosis is the process of infiltration of lipid into both the inter- and intra-muscular compartments and, similarly to sarcopenia, has been associated with worse outcomes in both oncological and non-oncological patients [12, 13].

Previous studies have examined the role of body fat analysis in association with outcomes of IBD patients but few studies examining the role of muscle mass and muscle quality have been conducted [14–16]. The aim of this study was to investigate sarcopenia and myosteatosis with respect to postoperative outcomes in patients undergoing surgical resection for IBD.

## Methods

### Patients

This study was conducted in accordance with STrengthening the Reporting of OBservational studies in Epidemiology (STROBE) guidelines [17]. Ethical approval was granted by the hospital Institutional Research Ethics Review Board. All consecutive patients who underwent a colonic resection for IBD, between 2009 and 2016 in a tertiary referral colorectal surgery service, were retrospectively identified from a prospectively maintained database. Patient medical records were analysed to obtain baseline demographics, including diagnosis, body mass index (BMI), presence of comorbidities (scored according to the American Society of Anesthesiologist [ASA]), preoperative medications, and smoking status. The Charlson Comorbidity Index (CCI) [18], which is predictive of 10-year survival and the neutrophil-lymphocyte ratio (NLR) were calculated for all patients. Perioperative haematological and biochemical levels were recorded. All surgeries were carried out by a single colorectal surgeon specialised in IBD treatment. The type of abdominal access and extent of surgical resection were documented from the operative note. Postoperative complications were graded according to the Clavien-Dindo classification system [19]. Intra-abdominal leaks were classified on the basis of their management [20]. A leak requiring action by surgery or interventional radiology was classified as clinically relevant.

### CT protocol, scan selection and image analysis

The hospital information technology system was searched for perioperative CT of the abdomen and pelvis in these patients. CT scans within 6 months prior to surgery or in the first postoperative month were selected. All patients underwent a CT scan of the abdomen and pelvis with a standardised protocol using the following parameters: scan range encompassing the lung bases to the pubic symphysis; 0.625-mm slice acquisition thickness; intravenously administered contrast (Iohexol, Omnipaque 300, GE Healthcare, Waukesha, WI, USA) delivered at 2.5 mL/s and imaged in the portal venous phase; 1.5 L of positive oral contrast (2% Gastrografin, Bracco Diagnostics Inc., Princeton, NJ, USA); tube voltage of 120 kVp; automated tube current modulation resulting in a variable current with a minimum of 50 mA and a maximum of 350 mA; gantry rotation time of 0.8 s; noise index 38. All CT images were acquired using a single 64-slice multi-detector row CT scanner (General Electric Lightspeed VCT-XTe, GE Healthcare, GE Medical Systems, Waukesha, WI, USA).

Preoperative scans were preferentially selected. When these were unavailable, the earliest postoperative scans were used. The CT scans were analysed using Osirix version 5.6.1 open source software (32-bit; http://www.osirix-viewer.com).

The cross-sectional skeletal muscle area (cm^3^) was measured, as previously described [11, 21]. Briefly, two sequential scans at the level of the third lumbar vertebra, in which both transverse processes were visible, were selected. The grow/regrow tool was used to measure the skeletal muscle area on these axial slices by a reviewer blinded to the patients’ demographics. The skeletal muscles included were the psoas, paraspinal and abdominal wall muscles. The threshold range for skeletal muscle was from -30 to +150 Hounsfield Units (HU) [22, 23].

**Fig. 1:**
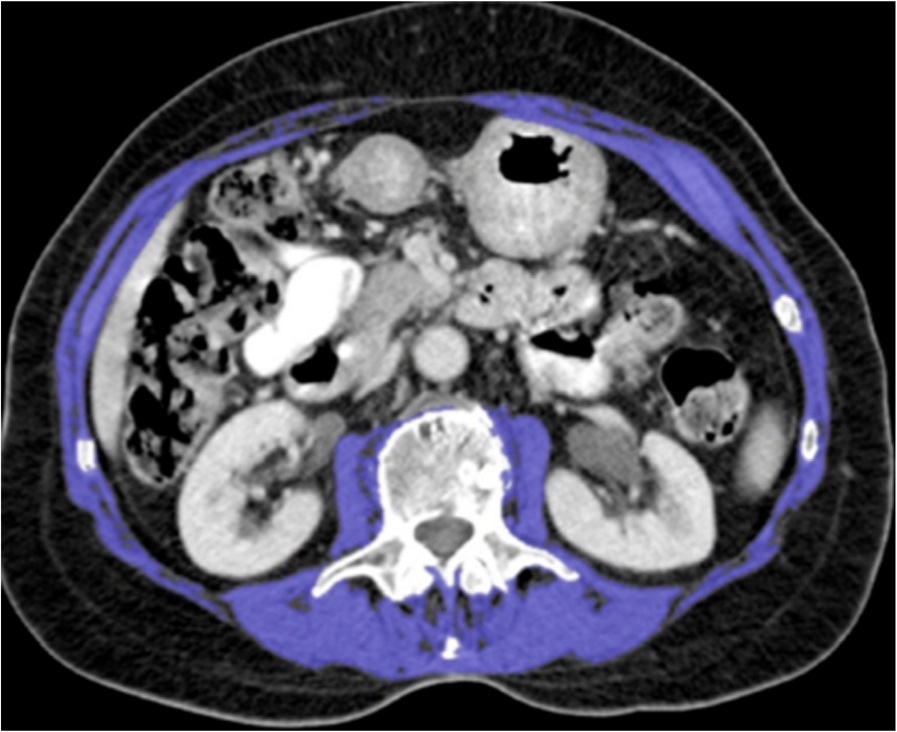
Example of computed tomography (CT) image analysis methods on Osirix version 5.6.1 open source software

The average muscle mass area of these two slices was used. The skeletal muscle area was normalised for height to produce the skeletal muscle index [24]. The specific cutoff values used for declaring a patient to be affected with sarcopenia were < 41 cm^2^/m^2^ in women, < 43 cm^2^/m^2^ in men with a BMI < 25 kg/m^2^, and < 53 cm^2^/m^2^ in men with a BMI ≥ 25 kg/m^2^, as published by Martin et al. [11]. The cutoff values for myosteatosis were < 41 HU in men or women with a BMI < 25 kg/m^2^ and < 33 HU in men or women with a BMI ≥ 25 kg/m^2^ [12]. An example of the methods employed for image analysis is demonstrated in Fig. 1.

### Statistical analysis

Statistical analysis was carried out using SPSS v20.0 software (SPSS Inc., Chicago, IL, USA). Normality was assessed using the Kolmogorov-Smirnov test. The independent *t* test and Mann-Whitney *U* test were used to compare continuous variables. The χ^2^ and Fischer’s exact tests were used to compare categorical variables. The impact of sarcopenia and myosteatosis was analysed by univariate regression analysis. A *p* value lower than 0.05 was used as the level of significance.

## Results

Ninety-six patients who had had surgery for IBD over the specified time period were identified from the database but 19 patients were excluded due to the lack of a suitable CT scan. These 77 patients were included in the study, 46 of them being male. Patient demographics, clinical indices, disease location/behaviour and pathological data are summarised in Table 1.

**Table 1:** Clinical indices and pathological data of patients stratified by the presence of preoperative sarcopenia

	All patients (*n* = 77) *n* (%)	No sarcopenia (*n* = 47) *n* (%)	Sarcopenia (*n* = 30) *n* (%)	*p*-value
Age, years (range)	42 (20–80)	41 (21–74)	43(20–80)	0.538^a^
Gender				
Male	46 (60)	31 (66)	15 (50)	0.124^b^
Female	31 (40)	16 (34)	15 (50)	
Diagnosis				
Ulcerative colitis	21 (27)	15 (32)	6 (20)	0.504^c^
Crohn disease	52 (68)	30 (64)	22 (73)	
Indeterminate colitis	4 (5)	2 (4)	2 (7)	
Location (Crohn)				
L1, ileal	14 (27)	9 (30)	5 (23)	0.443
L2, colonic	37 (71)	21 (70)	16 (73)	
L3, ileocolonic	1 (2)	0 (0)	1 (4)	
Behaviour (Crohn)				
B1, non-stricturing, non-penetrating	11 (21)	6 (20)	5 (23)	0.047
B2, stricturing	19 (37)	15 (50)	4 (18)	
B3, penetrating	22 (42)	9 (30)	13 (59)	
P, perianal disease	0 (0)	0 (0)	0 (0)	
Body weight, kg (range)	73 (32–129)	76 (53–118)	70 (32–129)	0.170^a^
BMI, median (range)	24 (16–37)	24 (19–33)	21 (16–37)	0.037^d^
BMI categories				
Underweight	5 (7)	0 (0)	5 (17)	0.028^c^
Normal	44 (57)	28 (60)	16 (53)	
Overweight	12 (16)	8 (19)	3 (10)	
Obese	16 (21)	10 (21)	6 (20)	
ASA physical score				
Grade 1	13 (17)	9 (19)	4 (13)	0.681^c^
Grade 2	49 (64)	30 (64)	19 (63)	
Grade 3	14 (18)	8 (17)	6 (20)	
Grade 4	1 (1)	0 (0)	1 (3)	
Charlson Comorbidity Index				
0	35 (45)	20 (43)	15 (50)	0.214^c^
1	20 (26)	15 (32)	5 (17)	
2	11 (14)	5 (11)	6 (20)	
3	3 (39)	3 (6)	0 (0)	
4	2 (26)	0 (0)	2 (7)	
5	4 (52)	2 (4)	2 (7)	
6	1 (1)	1 (2)	0 (0)	
7	1 (1)	1 (2)	0 (0)	
Smoking status				
Non-smoker	49 (64)	29 (62)	20 (67)	0.558^b^
Ex-smoker	15 (19)	11 (23)	4 (13)	
Smoker	13 (17)	7 (15)	6 (20)	
Preoperative steroid therapy				
No	35 (46)	22 (47)	13 (43)	0.475^b^
Yes	42 (55)	25 (53)	17 (57)	
Preoperative immunosuppression				
No	43 (56)	23 (49)	20 (67)	0.098^b^
Yes	34 (44)	24 (51)	10 (33)	
Preoperative anticoagulation				
No	73 (95)	45 (96)	28 (93)	0.509^c^
Yes	4 (5)	2 (4)	2 (7)	
Haemoglobin, male (range)	12 (10.5–13.6)	12.6 (10.5–13.7)	11.6 (10.3–12.3)	0.122
Haemoglobin, female (range)	11.6 (10.5–13.6)	12.8 (11.4–13.8)	11.0 (9.8–12)	0.024
White cell count (cells × 10^9^/L)	10.63 (3.7–28.1)	10.88 (5.1–28.1)	10.26 (3.7–19.6)	0.500^d^
Neutrophil-lymphocyte ratio	7.1 (1.09–26.81)	6.77 (1.09–22.65)	8.02 (1.11–26.81)	0.901^d^
Albumin (g/L)	34.9 (17–51)	35.28 (20–48)	33.52 (17–51)	0.283^d^
C-reactive protein (mg/L)	53.04 (0–547)	46.16 (0–374.7)	63.26 (0–547)	0.252^d^

*BMI* Body Mass Index, *ASA* American Society of Anesthesiologists

^a^ Independent *t* test, ^b^ χ^2^ test, ^c^ Fischer’s exact test, ^d^ Mann-Whitney *U* test

Of the 77 included patients, 30 (39%) were shown to be affected by sarcopenia and 26 (34%) by myosteatosis. Of the 52 patients (68%) diagnosed with Crohn disease, 22 had sarcopenia, 30 did not. Of the 21 patients (27%) diagnosed with ulcerative colitis, 6 had sarcopenia and 15 did not. There were 4/77 patients (5%) with a diagnosis of indeterminate colitis, of whom two had sarcopenia and two did not.

Stratification of patients based on the presence or absence of sarcopenia demonstrated that only patient weight and BMI were statistically different between the groups; patients with sarcopenia had a lower weight and BMI (*p* = 0.013 and *p* = 0.025, respectively). There was no difference in patient comorbidity profiles, as assessed by the ASA and CCI scores (*p* = 0.681 and *p* = 0.214). Of the 77 patients, 42 (55%) received preoperative steroid therapy and 34 patients (44%) had been receiving immunosuppressive therapy prior to surgery. While markers of inflammation, including the mean white cell count and C- reactive protein, were elevated, there was no difference between the groups (*p* = 0.500 and *p* = 0.252, respectively). Neither was a difference observed in white cell titres or haemoglobin levels.

Surgical details, including indications for surgery, are summarised in Table 2. No patients had perianal disease. Sixty percent of patients had emergency surgery. No difference was demonstrated between the patients with sarcopenia and those without in relation to the extent of surgical resection. Patient outcomes stratified by the presence of sarcopenia are presented in Table 3.

**Table 2:** Operative details stratified by sarcopenia

	All patients (*n* = 77) *n* (%)	No sarcopenia (*n* = 47) *n* (%)	Sarcopenia (*n* = 30) *n* (%)	*p*-value
Elective surgery				
Yes	31 (40)	19 (40)	12 (40)	0.581^a^
No	46 (60)	28 (60)	18 (60)	
Indication for surgery				
Stage 2 surgery for IPPA	6 (8)	3 (6)	2 (7)	0.850
Stricture	19 (24)	13 (28)	6 (20)	
Fistula	9 (12)	6 (13)	4 (13)	
Medically refractory disease	33 (43)	18 (38)	15 (50)	
Perforation	10 (13)	7 (15)	3 (10)	
Type of surgery				
Open	18 (23)	7 (15)	11 (37)	0.153^b^
Laparoscopic	52 (68)	35 (75)	17 (57)	
Laparoscopic-assisted	3 (4)	2 (4)	1 (3)	
Open → laparoscopic	4 (5)	3 (6)	1 (3)	
Extent of surgery				
Completion proctectomy, ileal pouch anal anastomosis and end-ileostomy	5 (7)	3 (6)	2 (7)	0.557^b^
Panproctocolectomy and end-ileostomy	6 (8)	3 (6)	3 (10)	
Sigmoid colectomy	6 (8)	5 (11)	1 (3)	
Ileocecectomy	14 (18)	9 (19)	5 (17)	
Anastomosis revision	1 (1)	1 (2)	0 (0)	
Right hemicolectomy	11 (14)	8 (17)	3 (10)	
Subtotal colectomy and end-ileostomy	27 (35)	15 (32)	12 (40)	
Left hemicolectomy and defunctioning loop ileostomy	1 (1)	1 (2)	0 (0)	
Completion proctectomy and end-ileostomy	3 (4)	1 (2)	2 (7)	
Ileorectal anastomosis	2 (3)	0 (0)	2 (7)	
Anterior resection and end-colostomy	1 (1)	1 (1)	0 (0)	

Elective surgery = planned hospital admission for surgery, non-elective surgery = non-planned surgical intervention. *IPPA* ileal pouch anal anastomosis

^a^ χ^2^ test, ^b^ Fischer’s exact test

**Table 3:** Analysis of hospital outcomes by sarcopenia

	All patients (*n* = 77) *n* (%)	No sarcopenia (*n* = 47) *n* %	Sarcopenia (*n* = 30) *n* %	*p*-value
Major complication (Clavien-Dindo ≥ 3a)	16 (21)	10 (21)	6 (20)	0.566^a^
Major intra-abdominal leak	11 (14)	7 (15)	4 (13)	0.564^c^
Length of hospital admission (days) median (IQR)	10 (8–15)	10 (8–14)	10.5 (7–18)	0.741^b^
Hospital readmission < 30 days	10 (14)	3 (6)	7 (23)	0.030^c^

*IQR* interquartile range

^a^ χ^2^ test, ^b^ Mann-Whitney *U* test, ^c^ Fischer’s exact test

The median length of hospital stay was 10 days (interquartile range 8–15). There was no significant difference in median length of hospital admission between patients with sarcopenia and those without (10 days versus 10 days, *p* = 0.741). There was a significant difference in the length of hospital stay between patients with myosteatosis and those without (13 days versus 9 days, *p* = 0.039). Table 4 stratifies the outcomes by the presence of myosteatosis.

**Table 4:** Analysis of hospital outcomes by myosteatosis

	All patients (*n* = 77) *n* (%)	No myosteatosis (*n* = 51) *n* (%)	Myosteatosis (*n* = 26) *n* (%)	*p*-value
Major complication (Clavien-Dindo ≥ 3a)	16 (21)	9 (18)	7 (27)	0.254^a^
Major intra-abdominal leak	11 (14)	5 (10)	6 (23)	0.111^a^
Length of hospital admission, days (median, IQR)	10 (8–15)	10 (7–13)	13 (9–24)	0.039^b^
Hospital readmission < 30 days	10 (13)	3 (6)	7 (27)	0.011^c^

*IQR* interquartile range

^a^ *χ*^2^ test, ^b^ Mann-Whitney *U* test, ^c^ Fischer’s exact test

Of 77 patients, 10 (14%) required hospital readmission within 30 days of surgery. There was a statistically significant difference in the number of hospital readmissions between the sarcopenia and the non-sarcopenia groups (*p* = 0.030). Patients with myosteatosis also required readmission more frequently (*p* = 0.011).

As sarcopenia and myosteatosis were associated with hospital readmission, we further analysed the strength of this finding. Univariate and multivariate regression analysis was used to identify factors associated with hospital readmissions (Table 4). Sarcopenia and myosteatosis were significant risk factors for readmission on univariate analysis (odds ratio [OR] = 4.778, *p* = 0.034 and OR = 6.451, *p* = 0.012, respectively). Only the presence of myosteatosis remained statistically significant on multivariate regression analysis (OR = 4.802, *p* = 0.043) (Table 5).

**Table 5:** Univariate and multivariate regression analysis for risk factors of readmission

	Univariate analysis			Multivariate analysis		
	OR	95% CI	*p*-value	OR	95% CI	*p*-value
Age	0.989	0.943–1.037	0.647			
Gender (male)	1.462	0.384–5.560	0.578			
Diagnosis						
Ulcerative colitis	1		1			
Crohn	1	0.232–4.310	0.999			
Indeterminate colitis	0.00	0.00–infinity	0.000			
BMI category						
Underweight	1					
Normal	0.848	0.082–8.792	0.890			
Overweight	0.000	0.00–infinity	0.999			
Obese	0.615	0.044–8.703	0.719			
ASA physical score ≥ 3	0.000	0.000–infinity	0.999			
Charlson Comorbidity Index ≥ 3	0.905	0.099–8.248	0.929			
Smoking status						
Non-smoker	1					
Ex-smoker	2.150	0.448–10.312	0.339			
Smoker	1.911	0.319–11.450	0.478			
Neutrophil-lymphocyte ratio > 5	1.929	0.496–7.5	0.343			
Albumin > 35 g/L	0.606	0.156–2.357	0.606			
Preoperative steroid use	3.758	0.740–19.086	0.110			
Preoperative immunosuppression	0.756	0.194–2.935	0.686			
Emergency surgery	1.703	0.403–7.191	0.469			
Sarcopenia	4.778	1.121–20.361	0.034	3.251	0.707–14.951	0.130
Myosteatosis	6.451	1.495–27.829	0.012	4.802	1.053–21.889	0.043

*OR* odds ratio, *CI* confidence interval, *BMI* Body Mass Index, *ASA* American Society of Anesthesiologists

Of the 77 patients, 16 (21%) had a Clavien-Dindo complication score greater or equal to 3a (postoperative complication treated with intervention not requiring general anaesthesia), and 11 (14%) had an intra-abdominal leak requiring treatment with surgery or interventional radiology. Univariate and multivariate regression analysis were used to identify risk factors associated with significant hospital complications (Clavien-Dindo score ≥ 3a) (Table 6). On univariate analysis, age greater than 65 years, CCI ≥ 3 and NLR > 5 were significant risk factors for complications (OR = 6.444, *p* = 0.024, OR = 4.167, *p* = 0.039 and OR = 4.320, *p* = 0.021). Only the presence of a NLR > 5 remained statistically significant on multivariate regression analysis (OR = 4.489, *p* = 0.024).

**Table 6:** Univariate and multivariate regression analysis for risk factors of major complication

	Univariate analysis			Multivariate analysis		
	OR	95% CI	*p*-value	OR	95% CI	*p*-value
Age > 65 years	6.444	1.274–32.593	0.020	4.274	0.242–75.356	0.321
Gender (male)	0.420	0.122–1.449	0.170			
Diagnosis						
Ulcerative colitis	1					
Crohn	3.167	0.648–15.474	0.154			
Indeterminate colitis	3.167	0.215–46.726	0.401			
BMI category						
Underweight	1		0.631			
Normal	0.435	0.063–3.021	0.400			
Overweight	0.375	0.035–3.999	0.417			
Obese	0.214	0.021–2.187	0.194			
ASA physical score ≥ 3	2.318	0.660–8.138	0.189			
Charlson Comorbidity index ≥ 3	4.167	1.078–16.103	0.039	1.663	0.141–19.573	0.686
Smoking status						
Non-smoker	1		0.228			
Ex-smoker	0.684	0.131–3.578	0.653			
Smoker	2.778	0.734–10.513	0.132			
NLR category > 5	4.320	1.249–14.498	0.021	4.489	1.222–16.494	0.024
Albumin > 35 g/L	0.525	0.169–1.629	0.265			
Preoperative steroid use	1.510	0.488–4.676	0.474			
Preoperative immunosuppression	0.502	0.156–1.617	0.248			
Emergency surgery	2.382	0.690–8.226	0.170			
Sarcopenia	0.925	0.297–2.878	0.893			
Myosteatosis	1.719	0.557–5.304	0.346			

*ASA* American Society of Anesthesiologists, *BMI* Body Mass Index, *CI* confidence interval, *NLR* neutrophil-lymphocyte ratio, *OR* odds ratio

## Discussion

This study assessed postoperative outcomes, including length of admission, readmission rates and serious complications, in a cohort of patients with IBD undergoing resection surgery, stratifying patients based on the presence of sarcopenia and myosteatosis. The results add to existing evidence indicating that patients who suffer from IBD requiring resection surgery who also have perioperative sarcopenia, and in particular myosteatosis, require increased postoperative care. The importance of myosteatosis in relation to postoperative outcomes is gaining increasing recognition [25] and this study provides new evidence in this regard.

These findings may form the basis for further assessment of the timing of surgery in such patients, taking into consideration the influence of pre- and postoperative nutritional status. In fact, many of the surgeries in IBD patients are performed as emergencies and offer little potential to optimise nutritional status prior to surgery. Nevertheless, patients with myosteatosis, despite spending longer in hospital, were also more likely to require readmission.

It has been previously demonstrated that hospitalised patients with IBD have a greater protein-calorie deficiency compared with non-IBD patients [26]. Investigation of methods for improving the nutritional and functional status of IBD patients prior to discharge following resection surgery, or also efforts on an outpatient basis, would help reduce readmission rates. Increased home visits or earlier review at outpatient clinics could work in this direction.

Patient selection is a potential confounding factor. However, the incidence of sarcopenia and postoperative complications in the present study were within expected limits. For example, the incidence of sarcopenia in patients with IBD has been reported to range from 26 to 60% [27–29]. The incidence of sarcopenia in the present cohort was 39% and, therefore, within this range.

On the other side, patients with ulcerative colitis were reported to have a lower incidence of sarcopenia when compared with those with those with Crohn disease [30], a difference which could potentially affect outcomes. However, patients with Crohn disease accounted for the greater proportion of patients in the present paper.

Almost 60% of patients in the present study underwent emergency surgery, which would have the potential to increase complication rates. The incidence of postoperative complications in the present paper was 21%, which was also within expected practice as complication rates of up to 27% have been reported in IBD patients [6]. A recent study by Pederson et al. [31] reported a similar incidence of Clavien-Dindo type-3a complications (25%) and similar length of hospital admission (8 days) compared with the present study. Six patients (8%) required reoperation in the present study, which is again comparable with published data [4, 5, 31].

The present study demonstrated no significant difference in the incidence of type-3a or greater Clavien-Dindo complications between sarcopenia and non-sarcopenia patient groups. Other studies reported differed data in this regard. Zhang et al. [28] found a decreased incidence of complications in non-sarcopenia patients (2.3 compared with 15.7%). However, when comparing this data with our results, we should consider that the patients were ten years younger, the definition of sarcopenia was slightly different, and the prevalence of sarcopenia was more than double.

Patients with IBD are prone to preoperative sarcopenia and myosteatosis due to a combination of chronic illness, abdominal pain hindering oral intake, malabsorption and the effects of medications [32]. In addition, disease activity and the pro-inflammatory state of IBD patients was perioperatively attested to in the present study by the increased incidence of clinically relevant complications with a NLR greater than five. This has been noted previously in the setting of colorectal carcinoma [33]. The treatment of postoperative leaks is very challenging in IBD patients due to the frequent presence of a hostile abdominal status, resulting in a preference for non-surgical drainage by interventional radiology approaches [34].

The impact of sarcopenia and myosteatosis on postoperative outcome has been examined to a greater extent in patients with colorectal cancer; however, there is a substantial variability in study methodology and results. In some reports the presence of sarcopenia and myosteatosis failed to show an association with the occurrence of intra-abdominal leaks or abscesses in patients undergoing colorectal cancer surgical resection, which concurs with the results of the present study [11, 20]. On the other hand, Huang et al. [35] found an association between sarcopenia and postoperative complications: 10 of 17 patients with sarcopenia had a grade 2 or greater CCI complication following surgery for colorectal cancer. This cohort therefore included patients with less complex complications in comparison with the present study.

The aetiology of sarcopenia and myosteatosis in cancer, which contribute to the cancer cachexia state, may differ from that of a benign condition, such as IBD, in which the processes causing the altered inflammatory state are more likely to be reversible either medically by immunosuppression or by surgical means. In the setting of cancer, it is generally believed that treating the disease will reduce the pro-inflammatory drive, but this goal is often not achievable [36]. Recent papers have reported that recovery of skeletal muscle volume tends to follow after induction of infliximab in Crohn disease patients [37] or after colectomy in ulcerative colitis patients [38]. In the present study, there was no significant difference in the complication rates between patients with ulcerative colitis and those with Crohn disease.

The classification of patients as affected with sarcopenia has been re-examined in recent years. An initial study by Prado et al. [23] provided cutoff values calculated in a population of obese patients, which may have caused skewing of results when applied to the general population. A follow-up study by Martin et al. [12] provided updated cutoff values with more heterogeneity in the cohort. In the present study, no association was demonstrated between sarcopenia or myosteatosis and postoperative complications when both the cutoff values published by Martin et al. or the lowest gender-specific cutoff were used.

The advent of low-dose CT has assisted in the assessment of acutely unwell patients with IBD who are at increased risk of high cumulative radiation exposure from diagnostic imaging [39]. This is the first paper to examine the roles of both sarcopenia and myosteatosis, as measured by CT, in patients with IBD with respect to postoperative outcomes. The aetiology of the findings is less clear due to its retrospective design. The inclusion of a preoperative quality of life questionnaire and a short nutritional assessment questionnaire should be considered in a prospective study. Other methods of investigating the role of nutritional assessment of patients with IBD have also been described in the literature. The volumetric assessment of visceral fat has been shown to be associated with postoperative complications and this could be correlated with CT-derived data [40]. The addition of preoperative measurements of muscle function, such as grip strength, could also be considered as direct comparisons with studies of postoperative outcomes in older patients with colorectal cancer are likely to be limited [9, 35].

In conclusion, the present study demonstrated an association between myosteatosis and increased hospital stay in addition to an increased 30-day readmission rate for patients with IBD undergoing resection. Given the treatable nature of IBD, there is a potential for sarcopenia and myosteatosis to be assessed in terms of potentially modifiable risk factors for adverse postoperative outcomes and, therefore, for being the target of future therapeutic targets.

## Abbreviations

ASA: American Society of Anesthesiologists
BMI: Body mass index
CCI: Charlson comorbidity index
CI: Confidence interval
CT: Computed tomography
IBD: Inflammatory bowel disease
NLR: Neutrophil-lymphocyte ratio
OR: Odds ratio
